# Tooth mousse containing casein phosphopeptide-amorphous calcium phosphate prevents biofilm formation of *Streptococcus mutans*

**DOI:** 10.1186/s12903-021-01502-6

**Published:** 2021-03-19

**Authors:** Ronit Vogt Sionov, Danae Tsavdaridou, Muna Aqawi, Batya Zaks, Doron Steinberg, Miriam Shalish

**Affiliations:** 1 The Biofilm Research Laboratory, The Faculty of Dental Medicine, The Institute of Dental Sciences, The Hebrew University of Jerusalem, Jerusalem, Israel; 2 International Postgraduate Program, Department of Orthodontics, Hebrew University-Hadassah School of Dental Medicine, Jerusalem, Israel; 3 Department of Orthodontics, Hebrew University-Hadassah School of Dental Medicine, Jerusalem, Israel

**Keywords:** Casein phosphopeptide-amorphous calcium phosphate (CPP-ACP), Oral biofilm, *Streptococcus mutans*, Dental caries, GC Tooth Mousse®

## Abstract

**Background:**

*Streptococcus mutans* is a common cariogenic bacterium in the oral cavity involved in plaque formation. Casein phosphopeptide-amorphous calcium phosphate (CPP-ACP) has been introduced into tooth mousse to encourage remineralization of dental enamel. The aim of this research was to study the effect of tooth mousse containing CPP-ACP (GC Tooth Mousse®) or CPP-ACP with 0.2% fluoride (CPP-ACPF; GC Tooth Mousse Plus®; GCP) on *S. mutans* planktonic growth and biofilm formation.

**Methods:**

*S. mutans* was cultivated in the presence of different dilutions of the tooth mousse containing CPP-ACP or CPP-ACPF, and the planktonic growth was determined by ATP viability assay and counting colony-forming units (CFUs). The resulting biofilms were examined by crystal violet staining, MTT metabolic assay, confocal laser scanning microscopy (CLSM), and scanning electron microscope (SEM).

**Results:**

The CPP-ACP tooth mousse (GC) at a dilution of 5–50 mg/ml (0.5–5%) did not inhibit planktonic growth, and even increased the ATP content and the number of viable bacteria after a 24 h incubation. The same was observed for the CPP-ACPF tooth mousse (GCP), except for the higher concentrations (25 and 50 mg/ml) that led to a drop in the bacterial count. Importantly, both compounds significantly decreased *S. mutans* biofilm formation at dilutions as low as 1.5–3 mg/ml. 12.5 mg/ml GC and 6.25 mg/ml GCP inhibited biofilm formation by 90% after 4 h. After 24 h, the MBIC_90_ was 6.25 mg/ml for both. CLSM images confirmed the strong inhibitory effect GC and GCP had on biofilm formation when using 5 mg/ml tooth mousse. SEM images of those bacteria that managed to form biofilm in the presence of 5 mg/ml tooth mousse, showed alterations in the bacterial morphology, where the streptococci appear 25–30% shorter on the average than the control bacteria.

**Conclusion:**

Our data show that the tooth mousse containing CPP-ACP reduces biofilm formation of the cariogenic bacterium *S. mutans* without killing the bacteria. The use of natural substances which inhibit biofilm development without killing the bacteria, has therapeutic benefits, especially in orthodontic pediatric patients.

**Supplementary Information:**

The online version contains supplementary material available at 10.1186/s12903-021-01502-6.

## Background

Biofilms are formed when clusters of microorganisms adhere to surfaces and secrete extracellular matrix that assists the further attachment of additional microorganisms [1]. Oral biofilms can appear on teeth, mucosa, restorations, and orthodontic appliances, and play an important role in the etiology of caries, periodontal diseases, and candidiasis [2, 3]. *Streptococcus mutans *(*S. mutans*) is a facultative anaerobic Gram-positive bacterium that plays a major role in oral biofilm formation [4]. The bacterium utilizes sucrose to synthesize adhesive fructans and glucans by the respective enzymes fructosyltransferase (FTF) and glucosyltransferase (GTF) [5]. These extracellular polysaccharides (EPS) stick to surfaces, such as the tooth enamel, and together with glucan-binding proteins (GBPs), they act as binding sites for *S. mutans* and other microbes, thereby forming dental plaques composed of a complex microbe community embedded in an extracellular matrix [5]. Besides contributing to dental plaque formation, *S. mutans* is highly cariogenic in virtue of its ability to process sucrose from nutritional substances into organic acids that lower the pH within the biofilm, resulting in decalcification of the tooth enamel [6]. The virulence of *S. mutans* is further enhanced by its ability to survive in an acidic environment [6]. *S. mutans* embedded in the biofilm express different genes than those expressed in their planktonic form, enabling adaptation to the biofilm setting [7]. The sessile state of the bacteria in the biofilm makes them less sensitive to anti-bacterial agents [7].

The same principles of biofilm formation in the cariogenic process also apply to orthodontic patients. Orthodontic patients present an outstandingly challenging environment that include brackets, bands, and other potential surfaces for biofilm growth. These surfaces make it more difficult to achieve good oral hygiene, which favor the formation of oral biofilms [8–10]. As a result, orthodontic treatment may cause enamel demineralization, varying from white spot lesions to cavities, and soft tissue inflammation [11].

The nanocomplexes formed between casein phosphopeptides (CPPs) and amorphous calcium phosphate (ACP) were documented by E.C. Reynolds for three decades ago to have profound anti-cariogenic activity [12]. CPPs are phosphorylated casein-derived peptides made by proteolytic breakdown of the milk products α_S1_-, α_S2_-, and β-casein. The CPPs which contain the cluster sequence of Ser(P)-Ser(P)-Ser-(P)-Glu-Glu, stabilize nanoclusters of ACP, resulting in increased calcium phosphate levels in dental plaque [12]. The ability of CPP to buffer free calcium and phosphate ions enables ACP supersaturation relative to the tooth enamel, thus reducing demineralization and enhancing remineralization [12–16]. Thus, CPP-ACP alone or together with fluoride has been suggested to be a novel compound for remineralization of dental enamel, incorporated in varnishes, pastes, lozenges, and dentifrices [17–21].

Tooth Mousse® (MI Paste®) and Tooth Mousse Plus® (MI Paste Plus®) contain 10% casein phosphopeptide–amorphous calcium phosphate (CPP-ACP) without or with 0.2% NaF, respectively (produced by GC, Tokyo, Japan). These tooth mousse were made based on the innovative study of Reynolds et al*.* [22] who developed Recaldent® (CPP-ACP technology). Tooth Mousse Plus® contains 900 parts per million fluoride in a molar ratio with the calcium and phosphate of 5 calcium, 3 phosphate and 1 fluoride which was found to be the optimal ratio for generating fluorapatite [22]. The fluoride ions are known to bind to calcium and phosphate ions that are released upon enamel demineralization by plaque bacterial organic acids. Because of the more compact structure of fluorapatite than hydroxyapatite, the fluorapatite better resists acid attack and thus prevents demineralization [23]. The aim of this study was to investigate the effect of Tooth Mousse ("GC") and Tooth Mousse Plus ("GCP") on oral biofilms and oral bacteria in vitro.

## Methods

### Preparation of tooth mousse suspensions

Tooth Mousse® ("GC"; MI Paste, GC Corporation, Tokyo, Japan) and Tooth Mousse Plus® ("GCP"; MI Paste Plus) which contain CPP-ACP and CPP-ACPF, respectively, were resuspended in brain–heart infusion broth (BHI, Acumedia, Lansing, Michigan, USA) or BHI containing 2% sucrose (BHIS) to a concentration of 10%. Then serial dilutions were done in BHI for planktonic bacterial growth or BHIS for biofilm formation studies. The suspension of the tooth mousse in BHI and BHIS had a neutral pH of 7.

### Planktonic growth of *Streptococcus mutans*

For planktonic growth, an overnight culture of *S. mutans* UA159 was diluted to an optical density (OD) of 0.05 at 600 nm and incubated with different dilutions of the test compounds in 200 μl BHI in 96-flat bottom culture plates (Corning) at 37 °C in 95% air/5% CO_2_. The planktonic growth in the presence of the test compounds was compared to control bacteria grown in BHI medium. Different dilutions of the test compounds in the absence of bacteria were used to measure background signals.

### Microbial cell viability assay

The BacTiter-Glo™ kit (Promega, Madison, WI, USA) was used to quantify the ATP levels in untreated and treated cells according to the manufacturer's instructions. Briefly, 150 µl of each sample was mixed with 150 µl of the reagent in 96-white μClear flat bottom plates (Greiner Bio-One) for 5 min on an orbital shaker. Thereafter, the luminescence was recorded using the M200 Tecan microplate reader (Tecan Trading AG, Switzerland). Different dilutions of the test compounds in the absence of bacteria were used to measure background signals.

### Colony forming units (CFU)

The number of bacteria in the untreated and treated samples was determined by doing repeatedly tenfold serial dilutions in 1 ml BHI and seeding 100 μl of each dilution onto BHI agar plates that were incubated overnight at 37 °C in the presence of 95% air/5% CO_2_. After incubation, the number of colonies was counted using the ImageJ software. The following equation was used to calculate the CFU per well in the original sample: Number of colonies x dilution factor x original volume of sample.

### Biofilm formation by *Streptococcus mutans*

For biofilm formation, an overnight culture of *S. mutans* UA159 was diluted to an OD of 0.05 at 600 nm and incubated with different dilutions of the test compounds in 200 μl BHIS in 96-flat bottom culture plates (Corning) at 37 °C in 95% air/5% CO_2_. At the end of the incubation period, the biofilms were carefully washed with PBS or DDW and quantified by the assays described below. The biofilm formation in the presence of the test compounds was compared to control bacteria grown in BHIS medium. Different dilutions of the test compounds in the absence of bacteria were used to measure background signals.

### Crystal violet (CV) staining of biofilms

To quantify the resulting biofilm biomass, the washed biofilms were stained with 200 μl of a 0.1% crystal violet solution (1:4 dilution in DDW of the Gram's crystal violet solution, Merck) for 20 min at room temperature [24]. Thereafter the biofilms were washed twice with DDW and the stain dissolved in 200 μl of a 33% acetic acid solution. The OD at 595 nm was measured spectrophotometrically using the M200 Tecan microplate reader. Different dilutions of the test compounds in the absence of bacteria were used to measure background signals.

### Metabolic activity of the biofilms

The metabolic activity of the biofilms was examined using the MTT assay [25]. In brief, 50 μl of a 0.5 mg/ml (1.2 mM) solution of MTT (3-(4,5-dimethyl-2-thiazolyl)-2,5-diphenyl-2H-tetrazolium bromide) (Sigma, USA) was added to the biofilms, and after an 1 h incubation, 150 μl of PBS was added, the supernatant discarded, and the tetrazolium formed within the biofilms dissolved in 200 μl of dimethyl sulfoxide (DMSO). The OD at 570 nm was measured spectrophotometrically using the M200 Tecan microplate reader. Different dilutions of the test compounds in the absence of bacteria were used to measure background signals.

### FilmTracer™ SYPRO® Ruby biofilm matrix stain

The biofilm biomass was also measured using the FilmTracer™ SYPRO® Ruby biofilm matrix stain (Invitrogen, Molecular Probes, Eugene, OR). The washed biofilms were incubated with 100 μl of the reagent for 30 min at room temperature, followed by several washes with DDW. Thereafter the fluorescence of the biofilms was measured in the M200 Tecan microplate reader with excitation at 450 nm and emission at 610 nm.

### Confocal laser scanning microscope (CLSM)

The biofilms were stained with the SYTO 9/propidium iodide (PI) Live/Dead BacLight viability kit (Molecular Probes, Life Technologies, Carlsbad, California, USA) according to the manufacturer's instructions [26]. The SYTO 9 green fluorescence dye, which enters both live and dead bacteria, was visualized using 488 nm excitation and 515 nm emission filters. The PI red fluorescence dye, which only penetrates dead bacteria, was measured using 543 nm excitation and 570 nm emission filters. Thus, live bacteria fluoresce green light, while dead bacteria fluoresce both green and red light. The samples were visualized for thickness and bacterial vitality using the Nikon Yokogawa W1 Spinning Disk Microscope with 50 μm pinholes. The biofilm depth was assessed by capturing optical cross-sections at 2.5 μm intervals from the bottom of the biofilm to its top. Three-dimensional images of the formed biofilms were constructed using the NIS-Element AR software. This software was also used to analyze the fluorescence intensity of SYTO 9 and PI staining in each captured layers of the biofilms. The biofilms of treated bacteria were compared to control untreated bacteria.

### High resolution scanning electron microscope (HR-SEM)

Untreated and treated biofilms were fixed in 2% glutaraldehyde in DDW for 20 min, washed in DDW, air-dried, gold-coated and visualized using an analytical Quanta 200 Environmental High-Resolution Scanning Electron Microscope (EHRSEM) (FEI, Eindhoven, The Netherlands). The biofilm structure was observed in different regions, each with increasing magnifications.

### Statistical analysis

All experiments were performed in triplicates and repeated three times. Statistical analysis was performed using the Student t-test using the excel Microsoft software. Statistically significance was determined when the p value was less than 0.05.

## Results

### The diluted CPP-ACP tooth mousse (GC) did not inhibit planktonic growth of *S. mutans*

**Fig. 1:**
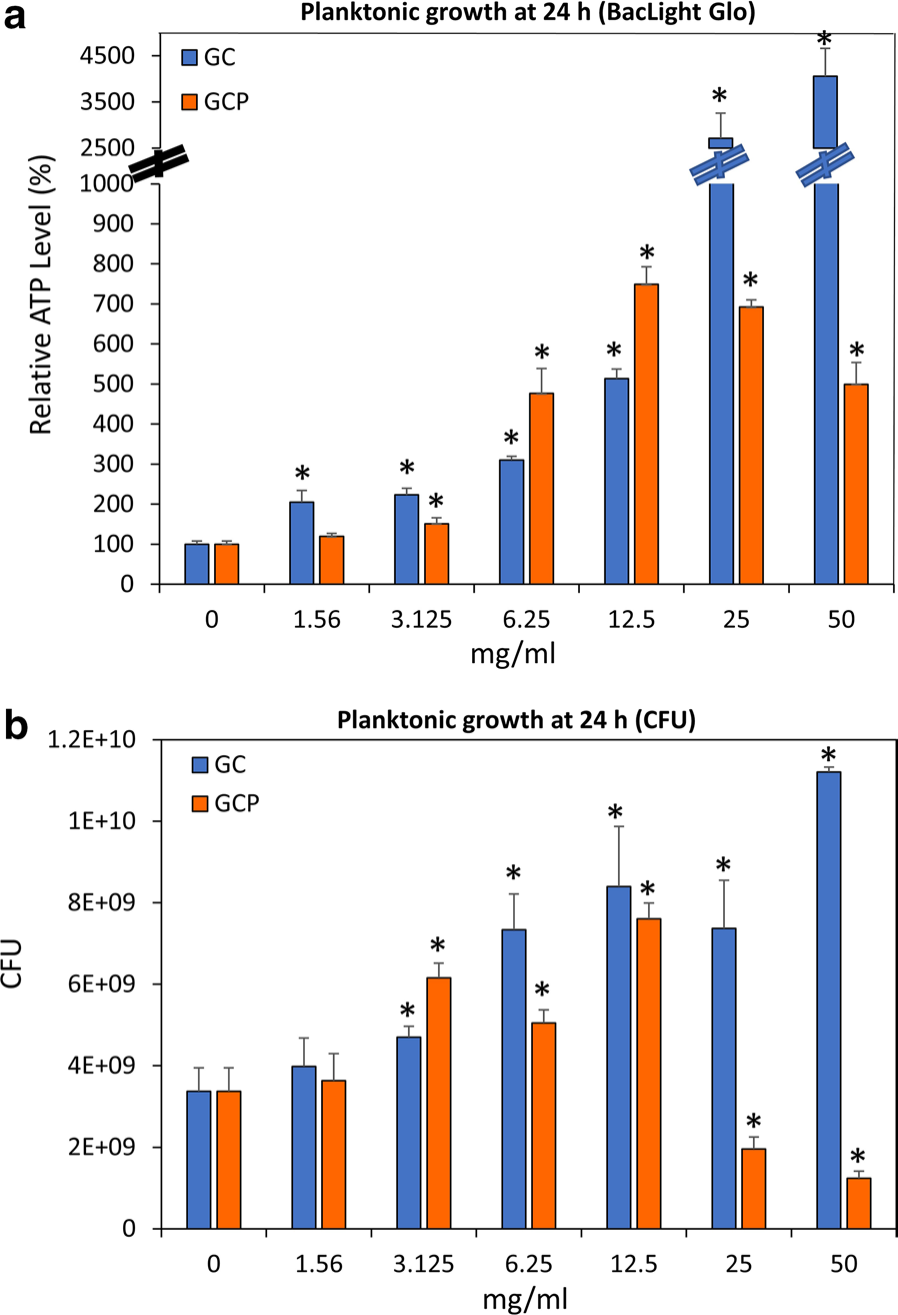
The effect of GC and GCP on planktonic growth of *S. mutans*. **a** The ATP content of *S. mutans* that have grown in the presence of various concentrations of suspended GC or GCP tooth mousse for 24 h. n = 3. **b** The CFU of *S. mutans* that have grown in the presence of various concentrations of suspended GC or GCP tooth mousse for 24 h. n = 3**.** * *p* < 0.05 in comparison to untreated bacteria

Our first question was whether the CPP-ACP-containing tooth mousse affects the planktonic growth of the cariogenic *S. mutans*. To this end, we incubated the bacteria with different dilutions of the tooth mousse for 24 h, and analyzed the bacteria viability using the BacTiter-Glo™ kit that measures the ATP content. Surprisingly, we observed that the diluted tooth mousse increased the ATP content in a dose-dependent manner (Fig. 1a). The diluted tooth mousse without bacteria gave only a relatively low signal with the reagent, suggesting that the luminescence originates from the bacteria in the sample. It could be that some of the ATP detected is due to ATP released from the bacteria that is retained by components of the tooth mousse. To figure out whether there is a true increase in the bacterial number, we determined the colony forming units (CFUs) in each of the samples. Indeed, we found a 1.4–3.3-fold increase in the bacterial count when *S. mutans* was exposed to 3–50 mg/ml GC tooth mousse (Fig. 1b). Similarly, we found an increase in the ATP content when using different dilutions of the CPP-ACPF tooth mousse (GCP) (Fig. 1a). When counting the live bacteria using the CFU assay, we noticed a 1.5–2.3-fold increase in the bacterial number with 3–12.5 mg/ml GCP, while 25–50 mg/ml GCP resulted in a 40–60% reduction in the viable bacteria after a 24 h incubation (Fig. 1b).

Since CPP is a tryptic digest of the milk protein casein and is composed of peptides and phosphate groups, it is likely that CPP might be a nutrient for *S. mutans*. To study this possibility, the bacteria were exposed to increasing concentrations of CPP, and the planktonic growth and ATP content were analyzed after 6 and 24 h (Additional file 1: Fig. S1). We observed that CPP treatment did not lead to an increased bacterial growth after 6 h, but caused a significant increase in the ATP content at doses of 5–50 mg/ml (Additional file 1: Fig. S1A). After 24 h incubation, there was 1.5-time more bacteria in samples treated with 10–50 mg/ml CPP than in control samples (Additional file 1: Fig. S1B). Again, we observed that CPP increases the ATP content per bacterium at concentrations 10–50 mg/ml when compared to control bacteria (Additional file 1: Fig. S1B). Thus, CPP may contribute to the increase in ATP content in bacteria exposed to GC and GCP tooth mousse, but it is likely that other components of the tooth mousse contribute to the increased proliferation of *S. mutans*.

### Both GC and GCP exerted strong anti-biofilm effects on *S. mutans*

**Fig. 2:**
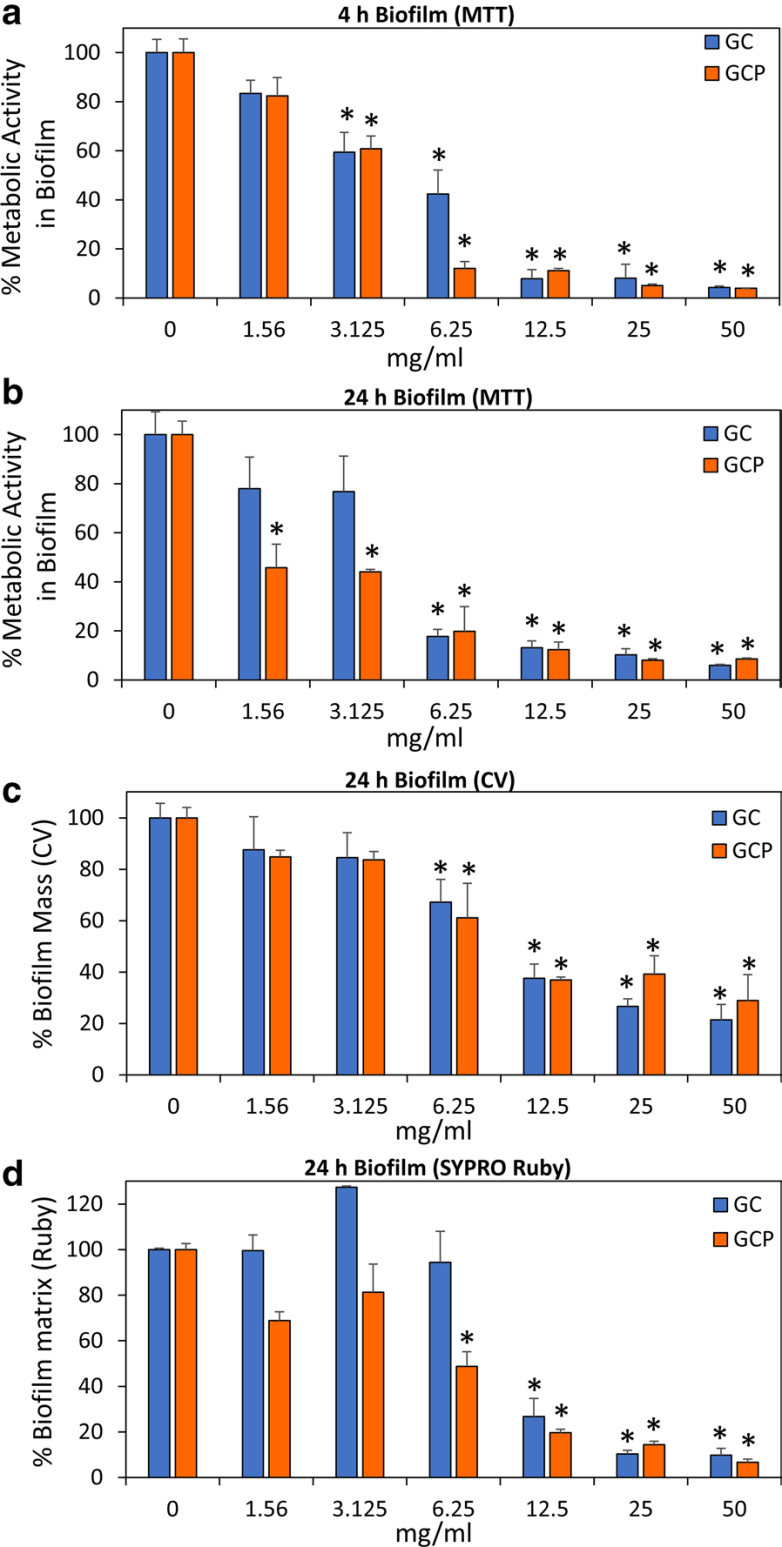
Anti-biofilm effect of GC and GCP on *S. mutans*. **a** Metabolic activity in *S. mutans* biofilms formed for 4 h in the presence of various concentrations of suspended GC or GCP tooth mousse as determined by the MTT assay. n = 3. **b** Metabolic activity in *S. mutans* biofilms formed for 24 h in the presence of various concentrations of suspended GC or GCP tooth mousse as determined by the MTT assay. n = 3. **c** Crystal violet staining of *S. mutans* biofilms formed for 24 h in the presence of various concentrations of suspended GC or GCP tooth mousse. n = 3. **d** FilmTracer™ SYPRO® Ruby biofilm matrix staining of *S. mutans* biofilms formed for 24 h in the presence of various concentrations of suspended GC or GCP tooth mousse. n = 3. * *p* < 0.05 in comparison to untreated bacteria

**Fig. 3:**
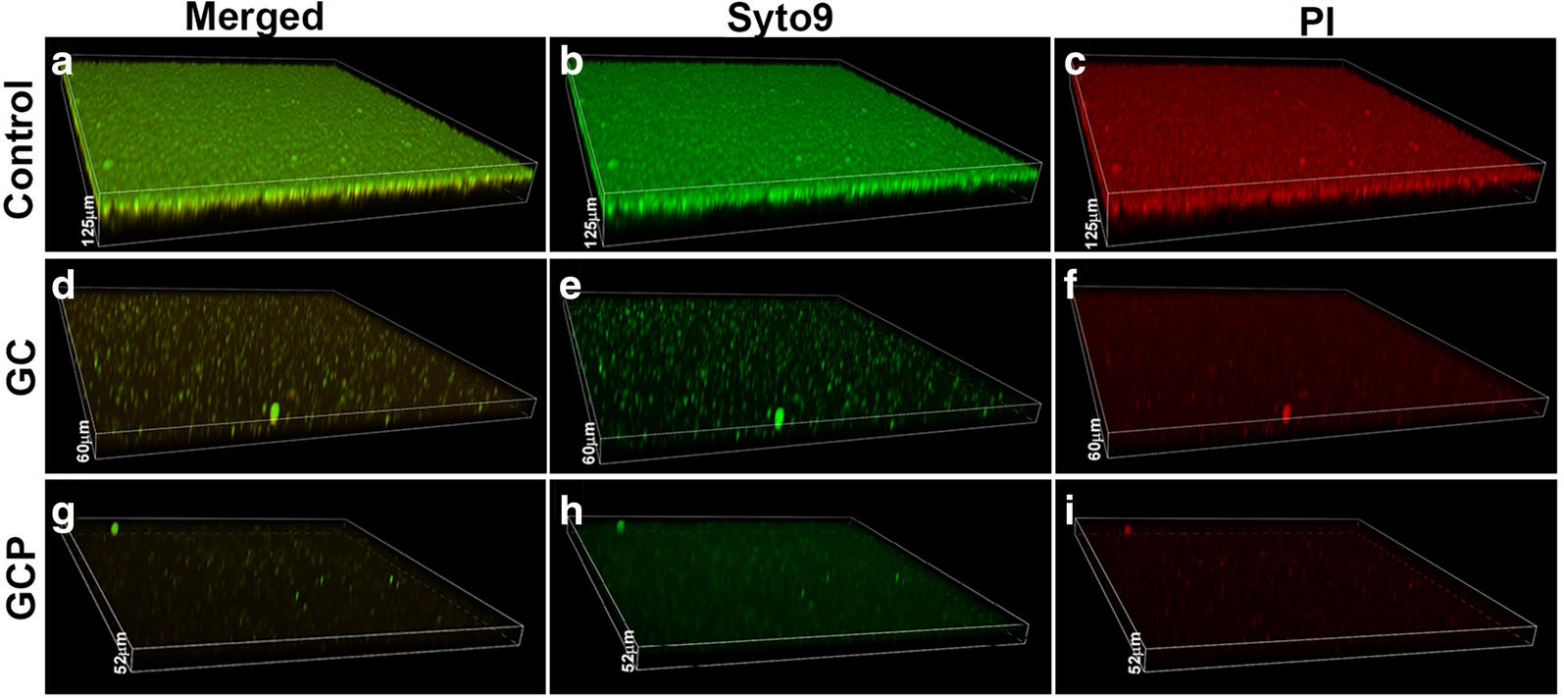
CLSM images of SYTO 9/PI-stained biofilms. Biofilms formed in the absence (**a–c**) or presence of 5 mg/ml GC (**d–f**) or GCP (**g–i**) for 24 h were washed in PBS and stained with SYTO 9 (green fluorescence) and PI (red fluorescence). The images are three-dimensional reconstructions of all layers captured using the NIS Element software. **a, d, g** are merged images of SYTO 9/PI. **b, e, h** are SYTO 9 staining. **c, f, i** are PI staining

**Fig. 4:**
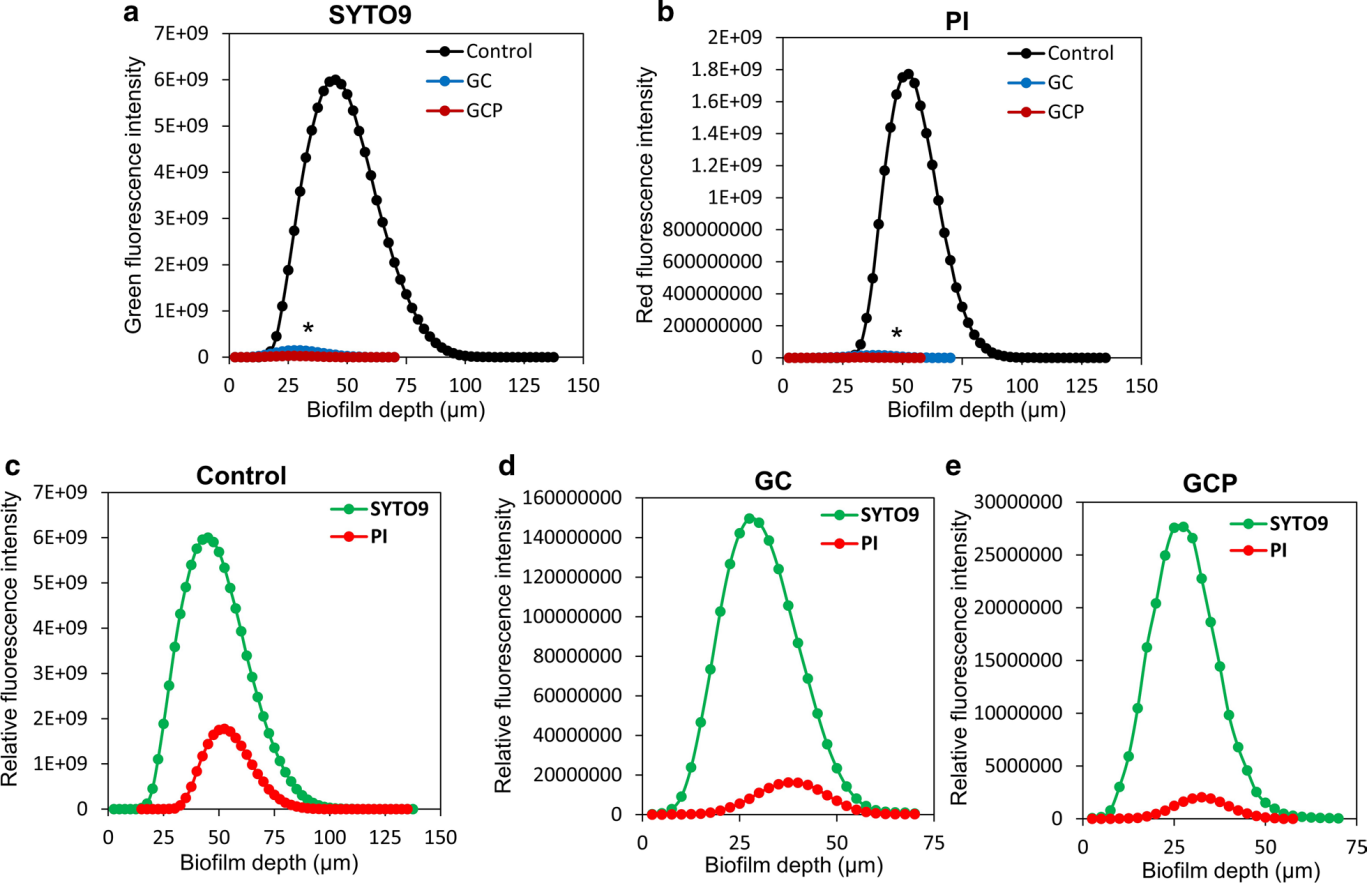
Quantification of the SYTO 9/PI staining of biofilms formed in the absence or presence of GC or GCP using the NIS Element Software**.** The average of calculations done on 4 different biofilms of each treatment group is presented. **a** Comparison of SYTO 9 staining of Control (black graph) versus GC (blue graph) and GCP (reddish brown graph)-treated biofilms. **b** Comparison of PI staining of Control (black graph) versus GC (blue graph) and GCP (reddish brown graph)-treated biofilms. **c–e** The comparison of SYTO 9 (green graph) and PI (red graph) staining of each treatment group. **c** Control biofilms. **d** GC-treated biofilms. **e** GCP-treated biofilms

We next examined the effect of GC and GCP on *S. mutans* biofilm formation. For this purpose, *S. mutans* was allowed to form biofilm in the presence of various dilutions of the tooth mousse, and the extent of biofilm formation was assayed after 4 h and 24 h. Already after 4 h, a dose-dependent reduction in the biofilm was observed as measured by the MTT metabolic assay (Fig. 2a). MBIC_90_ was 12.5 and 6.5 mg/ml for GC and GCP, respectively (Fig. 2a). Also, strong reduction of biofilm mass was observed after 24 h incubation with GC or GCP (Fig. 2b–d; Additional file 1: Fig. S2). The metabolic activity of the biofilms was reduced by 80% when exposed to 6.25 mg/ml of either GC or GCP (Fig. 2b). At the lower concentrations of 1.56 and 3.125 mg/ml, GCP reduced the metabolic activity of the biofilms by 50%, while GC had only a minor effect (Fig. 2b). When examining the biofilm biomass with crystal violet, a significant reduction of 60–80% in the biomass was observed at 12.5–50 mg/ml (Fig. 2c). In line with these findings, the FilmTracer™ SYPRO® Ruby biofilm matrix stain showed a dose-dependent reduction in the extracellular biofilm matrix (Fig. 2d). Again, GCP was more efficient than GC (Fig. 2d). The strong anti-biofilm effect of GC and GCP was further confirmed by confocal laser scanning microscopy using the live/dead BacLight viability kit (Fig. 3). GC and GCP at 5 mg/ml reduced the number of bacteria in the biofilm by 95–99% (Figs. 3d–f and g-i versus 3a–c; Fig. 4a, b). Also, the depth of the biofilm was reduced from 125–135 µm in control samples to 50–65 µm in samples exposed to GC or GCP (Figs. 3 and 4). The percentage of PI-positive bacteria dropped from 21.1 ± 4.3% in control samples to 9.8 ± 3.2% and 6.16 ± 1.2% in biofilms treated with GC and GCP, respectively (Fig. 4c–e). Altogether, our data suggest that the anti-biofilm effect is not due to killing of the bacteria, but rather a prevention of their adherence to the surface.

### GC- and GCP-treatment result in altered morphology of *S. mutans*

**Fig. 5:**
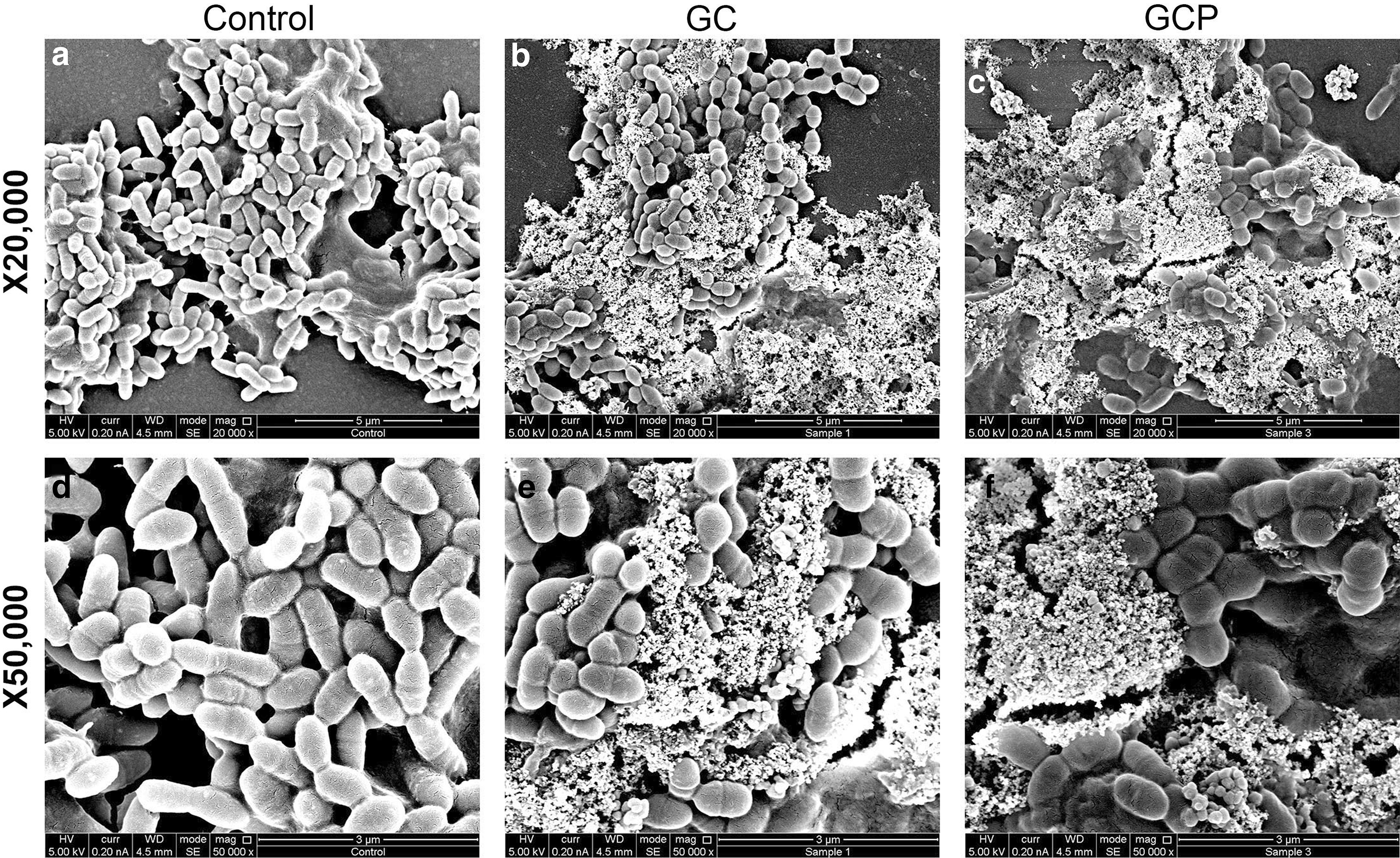
The effect of GC and GCP on the morphology of *S. mutans*. *S. mutans* was allowed to form biofilms for 24 h in the absence (**a, d**) or presence of 5 mg/ml GC (**b, e**) or GCP (**c, f**) for 24 h, and the morphology visualized by HR-SEM. Two different magnifications are shown

**Fig. 6:**
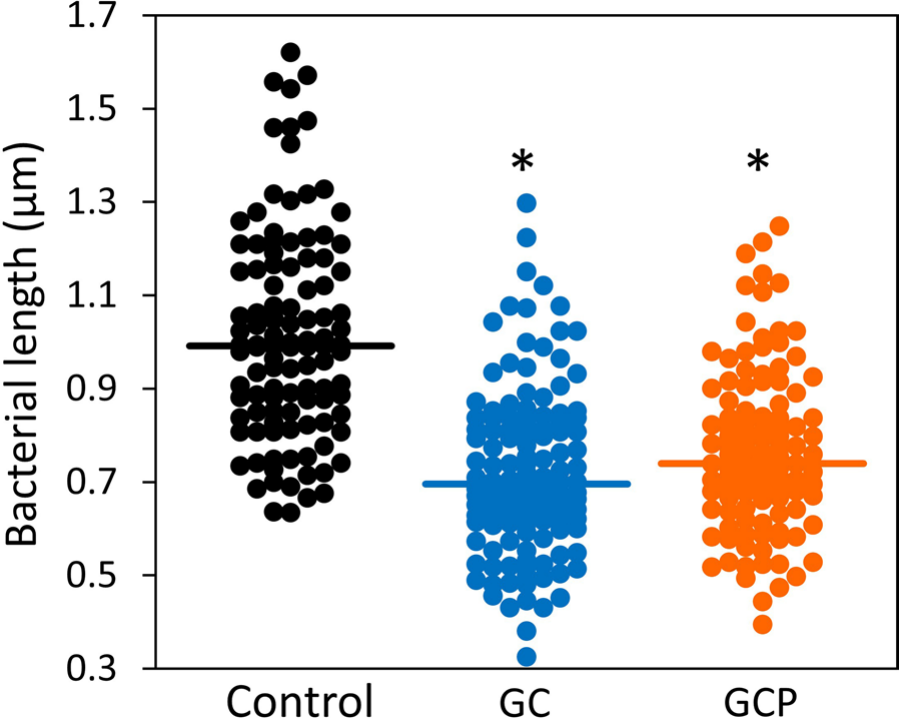
GC and GCP caused a reduction in the bacterial length of *S. mutans*. The length of around 100 bacteria were measured in 5 independent HR-SEM images of each treatment group presented in Fig. 5. * *p* < 0.05 in comparison to untreated bacteria

We next wanted to find out whether GC and GCP affect the morphology of the bacteria retained in the biofilms. For this purpose, biofilms formed in the presence of 5 mg/ml GC or GCP were inspected under a high-resolution-scanning electron microscope (HR-SEM) (Fig. 5). The control biofilm shows clusters and chains of bacteria embedded in extracellular matrix (Fig. 5a, d). The control bacteria appear as the classical *S. mutans* that show longer length than width (Fig. 5a, d) [27]. The bacteria in the biofilms of the GC- and GCP-treated samples are enwrapped in a precipitate of components likely from the tooth mousse that have adhered to the biofilm (Fig. 5b-c, e, f). The deposits resemble the structure of sol–gel-deposited calcium phosphate microstructures shown by Fotovvati et al. [28]. The bacteria in the treated biofilms appear shorter and have a more rounded up morphology (Fig. 5b, c, e, f). Systematic measurements of one hundred bacteria from five different images for each treatment group show that the length of the bacteria in the GC- and GCP-treated group was shortened by 25–30% (Fig. 6; *p* < 0.01).

## Discussion

Casein phosphopeptide-amorphous calcium phosphate (CPP-ACP) is a compound developed for the prevention of dental caries. This milk-derived agent enables remineralization and prevents demineralization as well as caries by generating a Ca/P reservoir on the teeth [22]. Additionally, CPP-ACP adheres to the salivary pellicle and thus may reduce the attachment of *S. mutans* [29, 30]. Based on these findings, these compounds have been incorporated into pastes and varnishes [31, 32]. Application of CPP-ACP paste in addition to regular oral hygiene protocol reduced demineralization (white spot lesions) in clinical studies [32–34].

Although several studies have examined the remineralization ability of CPP-ACP [16, 33, 34], only a few ones have assessed its anti-bacterial and anti-biofilm effectiveness [35–38]. In our study, we used various in vitro methods to explore the possible anti-bacterial/anti-biofilm properties of this compound toward the cariogenic *S. mutans*.

Our results demonstrate that GC tooth mousse containing CPP-ACP does not inhibit planktonic growth of S*. mutans* at any of the concentrations tested, and even enhanced the number of viable bacteria after a 24 h incubation. The simultaneous presence of fluoride ions in the GCP tooth mousse showed a similar growth-stimulating effect at higher dilutions (0.3–1.25%), while at lower dilutions (2.5–5%), a 40–60% reduction in the viable bacteria was seen that seemingly is due to the fluoride ions known to exert anti-microbial activities [39]. An interesting observation was the dose-dependent elevation in ATP content in the bacterial samples grown with increasing doses of GC and GCP. The relative increase in ATP content was higher than the relative number of live bacteria after a 24 h incubation, suggesting that components in the tooth mousse may affect the metabolism of *S. mutans,* resulting in elevated ATP production. We suspected that CPP could be the component, since it is composed of peptides and phosphate groups, which can be utilized by the bacteria as nutrition. Indeed, we observed that CPP significantly increased the ATP content of the bacteria, with only minor effect on the planktonic growth. It is likely that other components of the tooth mousse are responsible for the increased proliferation of *S. mutans*. It is notably that the increase in ATP content by CPP was modest (1.5–3 fold) in comparison to the extreme increase in ATP content (25–40-fold) in samples exposed to GC/GCP. One possibility for the high ATP content detected in the latter samples could be the binding of ATP released from the bacteria to the tooth mousse texture.

Importantly, both GC and GCP showed strong anti-biofilm activities even at high dilutions of more than 1 to a hundred, as demonstrated by reduced number of viable bacteria visualized by CLSM, reduced metabolic activity according to the MTT assay, and reduced crystal violet and SYPRO Ruby staining of the resulting biofilms. This finding accords with the study conducted by Dashper et al. [40], demonstrating that incorporation of 3% CPP-ACP into glass ionomer cements significantly reduced *S. mutans* biofilm development. We used a different biofilm model where the *S. mutans* was exposed to CPP-ACP-containing tooth mousse in suspension. Also, in this setting the CPP-ACP prevented the adhesion of *S. mutans* to the surface. Rahiotis et al. [36] applied the GC tooth mousse on orthodontic retainers and observed a delay in the biofilm formation in the presence of CPP-ACP. They further showed that CPP-ACP favored the nucleation and crystallization of calcium phosphates in the matured biofilms. Our SEM study show that both GC and GCP caused a deposit in the interspaces between the immobilized bacteria in the biofilm that resembles structures of calcium phosphate microcrystals [28]. This deposit barely adhered to the surface of the bacteria, suggesting that it causes unfavorable binding sites for the bacteria. The presence of this deposit on the surface might thus be a mechanism for preventing bacterial biofilm development. An anti-adhesion mechanism of CPP-ACP has also been proposed by Philip and Walsh [32]. Another interesting notation taken from the SEM images is the appearance of smaller and more rounded bacteria in the GC- and GCP-treated samples. The more rounded structure of the bacteria in the presence of the tooth mousse may be related to their reduced adherence to the surface, which is in contrast to the control bacteria that can spread on the surface. Actively dividing cells often show a more rounded morphology [41], such that the higher proliferation of *S. mutans* observed in the presence of GC/GCP might contribute to the altered morphology. A septum can be seen in many of the GC- and GCP-treated bacteria, suggesting that the bacteria are in division. The live/dead staining show that most of the GC and GCP-treated bacteria in the biofilms are alive (91–94%), which is in accordance with our finding that these compounds are not bacteriocidic. GCP that also contains fluoride, was more efficient than GC in preventing biofilm development, which can be explained by the contributing role of the fluoride ion in reducing biofilm formation of *S. mutans* [42]. Altogether, our data demonstrate that the GC and GCP tooth mousse prevent the adherence and biofilm development of the oral cariogenic *S. mutans*.

## Conclusions

Dentists and orthodontists seek to maintain good oral hygiene in their patients. Clinical trials show that the status of oral hygiene is enhanced once anti-bacterial mouth rinses are given as part of the oral hygiene protocol [43, 44]. The results of the present study suggest that CPP-ACP-containing products, in the form of mouthwash, could provide an anti-cariogenic effect based on the strong anti-biofilm activity against *S. mutans*. Such biofilm inhibition strategies are proposed to be suitable for orthodontic pediatric patients and for dental patients in general. It should, however, be kept in mind that dental caries might also be caused by other microbes besides *S. mutans* [3], and the efficacy of the CPP-ACP-containing products on preventing biofilm formation of these organisms needs to be proved. Further studies are required to demonstrate the microbial spectrum affected by CPP-ACP.

## Supplementary Information

**Additional file 1.** Supplementary Data.

## Data Availability

The datasets used during the present study are available from the corresponding author upon reasonable request.

## Abbreviations

ATP: Adenosine triphosphate
CFU: Colony forming unit
CLSM: Confocal laser scanning microscopy
CPP-ACP: Casein phosphopeptide-amorphous calcium phosphate
CV: Crystal violet
DDW: Doubled distilled water
GC: GC tooth mousse containing CPP-ACP
GCP: GC plus tooth mousse containing CPP-ACP and fluoride
MBIC: Minimum biofilm inhibitory concentration
MTT: 3-(4,5-Dimethyl-2-thiazolyl)-2,5-diphenyl-2H-tetrazolium bromide
PBS: Phosphate-buffered saline
HR-SEM: High resolution scanning electron microscope
