# Supplementary material for: Tooth mousse containing casein phosphopeptide-amorphous calcium phosphate prevents biofilm formation of Streptococcus mutans
Source: BMC Oral Health. 2021 Mar 19;21:136. doi: 10.1186/s12903-021-01502-6 (PMC7980609; doi:10.1186/s12903-021-01502-6)
Supplement: Supplementary file 1 — Additional file 1. Supplementary Data. [file 12903_2021_1502_MOESM1_ESM.pdf]

Supplementary Data

Tooth Mousse containing Casein Phosphopeptide-Amorphous Calcium Phosphate prevents Biofilm Formation of *Streptococcus mutans*

Ronit Vogt Sionov, Danae Tsavdaridou, Muna Aqawi , Batya Zaks, Doron Steinberg, and Miriam Shalish

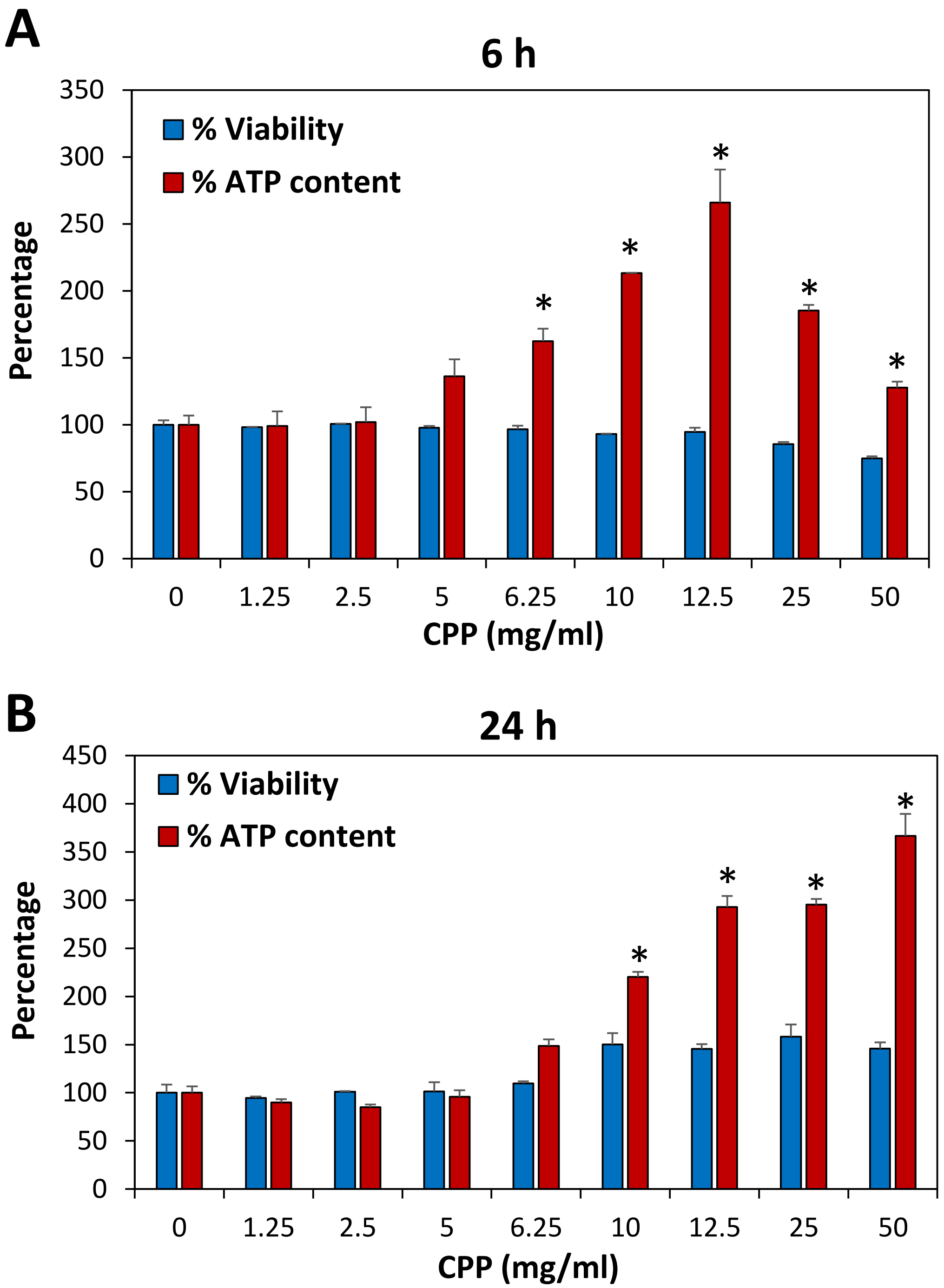

Suppl. Fig. 1. CPP increases the ATP content in *S. mutans*. *S. mutans* were grown in BHI in the absence or presence of increasing concentrations of CPP, and the OD of the planktonic growth (blue bars) and the ATP content (red bars) of the same cultures were measured at 6 h (**A**) and 24 h (**B**). * $p<0.05$.

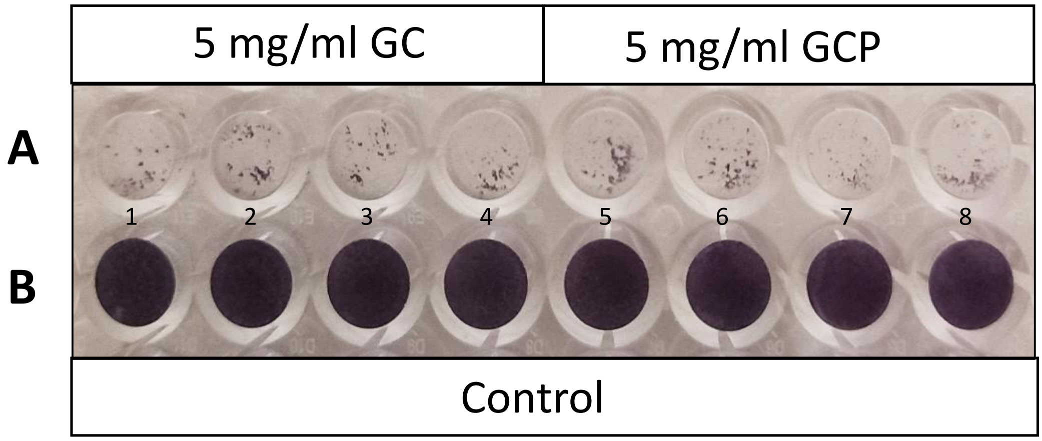

Suppl. Fig. 2. *S. mutans* was seeded in 96-flat bottom microwells in BHIS in the absence (Control, Row B wells 1-8) or presence of either 5 mg/ml GC (Row A wells 1-4) or GCP (Row A wells 5-8) for 24 h, and the metabolic activity in the resulting biofilms were stained with MTT.
